# Massage for Children Undergoing Hematopoietic Cell Transplantation: A Qualitative Report

**DOI:** 10.1155/2012/792042

**Published:** 2012-02-23

**Authors:** Sara L. Ackerman, E. Anne Lown, Christopher C. Dvorak, Elizabeth A. Dunn, Donald I. Abrams, Biljana N. Horn, Marcia Degelman, Morton J. Cowan, Wolf E. Mehling

**Affiliations:** ^1^Department of General Internal Medicine, The University of Califorina, San Francisco, CA 94143, USA; ^2^Alcohol Research Group (E.A.L.), Emeryville, CA 94608, USA; ^3^Department of Physiological Nursing, School of Nursing, The University of California, San Francisco, CA 94143, USA; ^4^Division of Blood and Marrow Transplant, Department of Pediatrics, The University of California, San Francisco, CA 94143, USA; ^5^Osher Center for Integrative Medicine, The University of California, San Francisco, CA 94115, USA; ^6^Department of Medicine, Hematology and Oncology, The University of California, San Francisco, CA 94115, USA; ^7^Department of Family and Community Medicine, The University of California, San Francisco, CA 94143, USA

## Abstract

*Background*. No in-depth qualitative research exists about the effects of therapeutic massage with children hospitalized to undergo hematopoietic cell transplantation (HCT). The objective of this study is to describe parent caregivers' experience of the effects of massage/acupressure for their children undergoing HCT. *Methods*. We conducted a qualitative analysis of open-ended interviews with 15 parents of children in the intervention arm of a massage/acupressure trial. Children received both practitioner and parent-provided massage/acupressure. *Results*. Parents reported that their child experienced relief from pain and nausea, relaxation, and greater ease falling asleep. They also reported increased caregiver competence and closeness with their child as a result of learning and performing massage/acupressure. Parents supported a semistandardized massage protocol. *Conclusion*. Massage/acupressure may support symptom relief and promote relaxation and sleep among pediatric HCT patients if administered with attention to individual patients' needs and hospital routines and may relieve stress among parents, improve caregiver competence, and enhance the sense of connection between parent and child.

## 1. Introduction

Therapeutic massage, a term that encompasses a wide variety of techniques of touch and tissue manipulation, has deep roots in the world's oldest medical practices, including both traditional Chinese medicine and Western medicine. In the late 19th century, a rift between massage and Western medical practice grew with the rise of scientific medicine, and physicians relinquished massage as a routine clinical practice. By the second half of the 20th century, massage had become professionalized and was increasingly associated with the alternative medicine movement [1].

More recently, a growing body of scientific literature on therapeutic massage—bolstered by its widespread popular use—has led to the reintroduction of various forms of massage as an adjunct to biomedical therapies. This shift is situated in growing popular and scientific interest in nonpharmacologic approaches to symptom management [2].

 Research on therapeutic massage has shown benefits in managing adult and pediatric patients' distress related to cancer and cancer treatment [3–7], and hematopoietic cell transplantation (HCT) [8, 9], although results are not consistent [10]. Acupressure massage has shown benefits for chemotherapy-related nausea [8, 11, 12], anxiety [13], and fatigue [8, 14]. In addition, performing massage on a family member with cancer has been shown to reduce anxiety and fatigue [9, 15] among caregivers, and to increase their sense of well being and confidence in managing their family member's symptoms [16–18].

 To date, most studies on therapeutic massage have measured predefined patient outcomes, usually medical and psychological symptoms. This growing body of research can be complemented by more in-depth investigations of patients' and caregivers' lived experiences of receiving and performing massage. Qualitative research has demonstrated, for example, that massage practices contribute to improvements in patient-caregiver relations [19] and to the “meaningful relief” of suffering among cancer patients [20]. Through open-ended interviews and close attention to the perceptions and interactions of participants, clinicians, and researchers, qualitative methods can examine aspects of massage practices that may go undetected by quantitative methods. This study focuses primarily on the perceptions and experiences of parent caregivers of pediatric HCT patients at an academic hospital. Parents living with and caring for a pediatric HCT patient typically spend weeks and even months in an isolated hospital room with filtered air and limited access to visitors. Parents are neither patient nor clinician; they are witness to their child's pain, suffering and confinement, as well as healing and resilience.

This study is one component of a mixed-method, randomized controlled pilot study introducing a combined Swedish and acupressure massage intervention in a pediatric HCT hospital unit. The overall aim of the pilot study was to assess whether conducting a study of such an intervention is feasible in the HCT unit, whether massage/acupressure alleviates patients' and parent-caregivers' distress and discomfort associated with HCT and accompanying chemotherapy, and to explore the effects of caregivers' experiences performing massage. Quantitative outcome measures of this feasibility study are reported separately [21]. The study described here examined caregivers' experiences learning to perform massage for their child and observing their child receive massage from a professional massage practitioner. Building on recent, more quantitatively oriented research assessing the effects of massage for pediatric HCT patients [6, 9], this study offers important new findings through the most in-depth, descriptive analysis to date of parent- and practitioner-provided massage for children undergoing HCT.

## 2. Methods

The massage intervention provided (1) practical, hands-on training for parents to provide massage/acupressure for their child; (2) professional practitioner-performed massages and acupressure treatments for children undergoing HCT. Professional massage practitioners provided up to three massages/acupressure sessions per week to the children during their entire hospital stay (days of hospitalization: median 37, range 23–110). They demonstrated massage/acupressure techniques to the parents for additional parent-provided massages for approximately ten minutes whenever the parent was present and amenable to it and provided a detailed hand-out with locations of and indications for specific acupressure points. The massage practitioners received training in several sessions with the research team (EAL, WEM) and received additional consultation from Traditional Chinese Medicine practitioners at the academic medical center. The massage intervention was a semistandardized integration of Swedish massage (gentle to moderately firm strokes, light pressure, holding touch to the back, shoulder girdle, hands, and legs) and acupressure based on traditional Chinese medicine using points on the feet, lower legs, wrist, and chest that are commonly used for nausea, pain and distress (9 points: PC6, ST36, LI4, LV3, BL62, KI6, SP6, HE7, CV17) [22]. Massage practitioners had more than ten years of experience each with Swedish massage and acupressure in a hospital setting. Variations in pressure, strokes, and massaged body areas were permitted within the frame of the intervention manual according to the child's needs and response. Symptom-specific acupressure points were selected according to patient needs and the massage protocol instructions. Foot massage (Swedish and acupressure) was routinely given for relaxation. In what follows, we will use the shorter term “massage” for the combination of Swedish massage elements, including foot massage, with acupressure as it was applied in this study. Massage duration was typically 10 to 30 minutes. Children wore hospital gowns during massage. Massage practitioners produced written reports on the type and duration of each massage, their impression of the child's response to the massage, and how they adapted their technique to accommodate this response.

 We decided to interview parents and massage practitioners exclusively in order to reduce the burden of research participation on the children. Data collection was restricted to the intervention arm using interviews with parents after hospital discharge, detailed hand-written notes by massage practitioners about each massage session, and interviews with two massage practitioners.

 Semistructured interviews with twelve mothers and three fathers were conducted by telephone approximately one week after hospital discharge. Each interview lasted approximately 30 minutes. Semistructured interviews with two massage practitioners were conducted by phone and lasted approximately 45 minutes. Interviews were conducted by two authors (ED and SA) who were not directly involved with the study intervention or the medical and nursing care on the unit. Practitioner interviews asked massage practitioners open-ended questions about their experiences performing massage for study participants, and their impressions of the children's experiences receiving massage and parents' experiences performing massage. The questions asked in the parent interviews were as follows.

“Can you tell us in a few words how it was for you  to learn some massage and to massage your child?”
“What was the best thing about the massage experience for you?”
“Was it possible for you to give massages?”

(*If yes:*) “How did that go for you and your child?”
(*If no:*) “What was the biggest barrier for giving a massage?”

“What was the best thing for you when you were giving your child a massage?”
“What do you think was the best thing about the massage experience  for your child?”
“Was it the same with the practitioner and with you?”
“What do you think was the hardest thing about the massage experience for your child?”
“What was the hardest thing about the massage experience for you?”
“What was the hardest thing about the massage study?”

### 2.1. Participants/Context

English-speaking children, 5 to 18 years of age, and their parents were invited to participate in the randomized controlled trial (RCT) as they were consecutively admitted to the transplant unit over a twelve month period (November 2008 to December 2009, patient characteristics in Table 1; additional details in [21]). Participation in the RCT was offered to child and parent during their preadmission consenting visit with a nurse manager and attending physician. No children over age 5 were excluded. Twenty-three children and their parents (rooming in with the child in the same hospital room) signed informed consent, enrolled and were randomized 2 : 1 to intervention versus control. The RCT was registered with clinicaltrials.gov NCT00843180. The qualitative study reported here included 15 of the 16 parents in the intervention arm. No payment was offered to participants in this group. Both the RCT and the qualitative study were approved by the university's Human Subjects Review boards.

 Children under age twelve signed assent forms, children twelve years and older signed consent forms, and parents signed consent for their own participation and their children under age 18. Seven child-parent dyads were assigned to the control arm, sixteen child-parent dyads to the intervention. One child subsequently declined all massages. The remaining 15 dyads are the subjects of this qualitative study (Table 1): eight were mother-son or father-daughter and seven were mother-daughter or father-son. Children in the intervention arm underwent autologous or allogeneic HCT for treatment of malignancies and other diseases listed in Table 1. Participants remained on the unit in their individual hospital rooms, behind double doors with high-level infection precautions. Massage group participants received 8.5 professional massages (median) during their hospitalization, at an average of 1.6 massages per week.

### 2.2. Analysis

Qualitative data analysis was conducted collaboratively by three of the authors (SA, EAL, WEM). The interpretive process was iterative and multistaged and included coding and thematic development [23, 24]. Data included transcripts of audio-recorded interviews, massage practitioners' written reports on massage sessions and study activities. First, interview recordings were listened to, and interview transcripts and massage therapists' report cards were read repeatedly, in order to form an overall impression of participants' and practitioners' experiences. Recurrent themes and patterns were identified in the data—particularly in terms of massage's effects on patients' and caretakers' experiences at the intersection of cognitive, affective, and physical states. Descriptive categories, or codes, were then developed for each emerging theme, and data fragments were systematically assigned codes. After the data were organized by code, key concepts were reworked through further discussion and analysis. This process included linking data extracts back to their original narrative context and conceptually situating ambivalent and contradictory statements.

## 3. Results

Three major themes were developed: (1) perceived benefits of massage for patients; (2) massage's effects on parents and family dynamics; and (3) impact of the timing and duration of massage therapy over the period of hospitalization. Each theme, along with related subthemes and examples, is described below. The voices of participants (parents of patients), massage practitioners, and researchers are included; their words are reported verbatim, revealing differing levels of English fluency among participants. Quotes are identified as follows:

P = parent of pediatric HCT patient,
R = research assistant/interviewer,
M = massage practitioner.

## 4. Parent-Perceived Benefits of Massage for Patients

Without exception, parents said that massage brought relief, comfort, and even pleasure to their children, although the effectiveness of massage in relieving specific treatment-related symptoms was variable among patients. The particular strength of the massage intervention appeared to be in promoting pleasurable sensations and a state of relaxation, with many children dozing off near the end of massage sessions. Most parents reported that their child looked forward to massages performed by parents and/or practitioners, and several parents continued to perform massage on their child after completion of the study. According to a massage practitioner, nurses on the transplant floor also described children's eagerness for the massage visits.

### 4.1. Symptom Relief

Parent caregivers reported that massage—performed by the professional massage therapist and/or a parent—provided relief from or support with symptoms, including nausea, pain, and inflammation.

#### 4.1.1. Pain

*(P6) We still use the pressure points. She loves the foot pressure points for the pain. She enjoys it.*
*(P17) I think it was very beneficial to have massage during the time when he was in a lot of pain and very uncomfortable. It was a good distraction and comforting.*

#### 4.1.2. Nausea

*(P1) It was amazing, especially with the nausea points. It worked.*

*(P20) Even though she's got headache or even though she's got vomiting, she wanted to have massage.*

#### 4.1.3. Inflammation

*(P21) When he had the joint inflammation, they were able to relieve–to help him through that.*

However, according to parents, not all patients found consistent symptom relief through massage.

*(P6) For pain it did not work as well, but the nausea—it really did, at times, alleviate all the nauseous feeling.*

*(P17) I cannot say that, you know, when I press on a certain area that it really made it [nausea] go away.*

### 4.2. Positive Feelings, Relaxation, and Sleep

Although relief from acute physical symptoms was reported by parents to be variable among patients, massage was uniformly associated with relaxation, comfort, positive physical sensation, and greater ease falling asleep. Whereas physical contact is often associated with uncomfortable treatments and procedures for children on the transplant unit, massage offered more pleasurable, calming sensations, which was seen by the massage providers as a way for children to “be in their bodies” instead of dissociating from them. A massage practitioner reported, for example, that a patient told her he felt like he was “floating on air” after massage.

* (P12) *[Massage]* make her more comfortable. At least make her have a better sleep. *
*(P7) The best thing about the whole experience was knowing that it helped him to relax.*
*(P14) Best thing is that it was making him relax, feel calm, and then he went to sleep.*
*(P21) He was really in a lot of pain, and for him to fall asleep during that 15 to 20 minutes was amazing… so that's–I took pictures [laughs].*
* (M) Some of the kids were trying not to be in their bodies because the whole thing was so unpleasant. The massage was a way for them to be in their bodies in a way that was pleasurable.*

### 4.3. Special Treatment

Parent caregivers often experienced massage as a practice situated outside of the transplant unit's routine activities, and as a kind of nonmedical therapy—or gift—that attended to the patient as a complex, feeling person, in contrast to the biomedical emphasis on treating disease as an entity belonging to the body and distinct from the person.

*(P21) It really is one of the few things outside of medicine that I saw work. You know, right before your eyes you can see the results.*
* (P20) I learned that giving a massage to my daughter was kind of changing atmosphere. It made more comfortable and safe…that was not the kind of giving medicine, but giving a kind of touch. There is big difference between medicine and massage.*
* (P13) When the therapist came in…she [the daughter] was getting the royal treatment… It was a person that was bringing peace to her versus an injection or taking blood from her. [The massage therapist] was not taking from her, but giving…I think it's just good for their soul…*

### 4.4. A Heightened Sense of Control

Hospital patients are subject to frequent and unannounced invasions of their privacy and bodies, and they often experience a sense of loss of control. This may be particularly true of pediatric patients, for whom cooperative decision making is limited. Massage sessions offered through this study, in contrast, took place only with patients' consent. Participation gave patients the opportunity to say “yes” or “no” and to shape the course of therapy, in a context in which their control over their environment and bodies is greatly diminished.

*(P12) Sometimes she do not want anybody to bother her…and I'm not bother her…only when she want it [massage] and she needs it.*

*(P22) Sometimes she did not want to be touched, so I would just leave her alone.*
* (M) Massage is the one thing in the hospital regimen that is voluntary, where the kids have the power to say “no.” It is the one time where how they feel is the most important thing. I think this made them feel empowered.*

### 4.5. Tailoring the Massage Protocol

Not only were patients able to choose whether to undergo a massage when a session was offered to them, at each session the patient was asked by the massage practitioner to describe in detail how he or she was feeling. Following a semistandardized protocol with specific instructions on how to choose acupressure points, practitioners would tailor the massage based on the patient's self-reported key symptoms, making adjustments throughout the session according to the patients' response. This process of tailoring within a given frame of techniques and specific acupressure points was described as essential to massage's efficacy by practitioners. Although parents were not asked directly about adapting massage techniques to their child's current physical and emotional state, their use of this approach was implicit in interview narratives.

*(M) Mom said they'd used the P6 point when she was feeling nausea earlier in the day and it had helped. Mom said she wished she would move around more so I showed them some gentle stretches she could do in bed- knees pulled up to belly and knees going to either side… so she could do ST36 point herself.*
*(M) I think it is really important…that the massage therapist be able to determine what the patient needs at the moment because their needs do change.*
* (P4) On last Sunday when he feel his body ached, so I just rub him. Most of the time he do not feel like getting massage.*

## 5. Massage's Effects on Parents and Family Dynamics

### 5.1. Performing Massage Improved Parents' Confidence as Caregivers

Most parents reported that they were able to learn massage techniques, and that they performed massage for their child intermittently or regularly—particularly parents of younger patients. Feelings of helplessness and anxiety are common among parents with hospitalized children, and parents expressed satisfaction and pride at being able to offer comfort and symptom relief to their children.

*(P21) It was great. It was one of the few things I could do to help him through everything he was going through.*
*(P23) It felt good because I was able to put her at peace, relax, help her to go to sleep, help her with the pain.*
*(P13) For me it was, as a parent, taking control of her pain and just providing the peace that she needs. So just creating an environment of peace that you normally do not find in a hospital…I was reassuring her that…it was going to be all right.*

### 5.2. The Social Effects of Massage

Parents reported that performing massage contributed to a heightened sense of intimacy and connection with their child.

*(P10) When I give her massage, I just feel closer to her. I feel we're like one.*

*(P13) When you massage someone, you're touching them, and you're loving them at the same time…it gives you the desire to love your child, to touch them, to let them know that you are there for them.*
* (R) What was the best thing for you when you were giving your child a massage?*
* (P3) Well, that bond that occurs between two people when there's comforting happening.*
*(P22) To be able to talk to her and touch her at the same time, and just talk about how she was feeling.*

At least two parents, however, reported that they did not readily learn or perform massage for their child, either due to the parent's perceived lack of competence or a missed opportunity for instruction in massage because she welcomed the opportunity for respite outside the hospital room when the massage practitioner arrived.

*(P6) It helped when the massage therapist would show me and do it on my child, and then I would do it with her there. After she left, I kind of forgot where those [pressure points] were.*
*(M) She seemed amenable to learning the points and doing massage, but in practice she wasn't often there when I came… Some of the parents used the time when I came as respite time to leave the room.*

Indeed, practitioner-provided massage offered parents a respite from caretaking and worry about their child, particularly since individual massage therapists became known and trusted by patients and parents over the course of the intervention. The knowledge that their children were experiencing comfort or pleasure provided stress relief for the parent and an opportunity to relax and take a break from the confines of the hospital room.

*(P4) Sometime when she's give him massage, I was like out for a walk. Sometime when the therapist come in, I was like tired and fall asleep and nap.*
*(P7) It was actually relaxing. Knowing that it was helping him to relax, then it also helps me to relax.*
*(M) It gave the kids a sense of nurturing when their parents were absent or too overwhelmed or exhausted to provide physical touch.*

Although most parents reported feeling closer to their child as a result of learning and performing massage, it is important to note that parent-child or broader social dynamics can also be a barrier to massage as a beneficial practice for children undergoing HCT. For example, one child mentioned to the professional massage practitioner that she preferred the professional massage to her father's massage, but felt uncomfortable communicating this to her father. Once this was revealed, the massage practitioner was able to help the father to improve his technique. Also, two teenage boys in this study declined to receive massage from parents or massage practitioners (all women), which may suggest possible discomfort with touch or embarrassment of female touch.

## 6. Impact of Timing of Massage and Length of Hospitalization

The timing of massage treatments was an important factor in parents' perception of the efficacy and desirability of massage. The concept of timing includes how a massage session fit into a patient's daily schedule, and whether massage was perceived as more or less beneficial at particular phases of an individual's journey through the transplant process. In addition, participants who remained hospitalized—and therefore enrolled in the study—for a longer period of time often moved from initial skepticism to becoming strong advocates for massage.

### 6.1. Fitting Massage into the Daily Clinical Routine

Patients on the unit follow a busy schedule of tests and treatments, and the degree to which massage was welcomed by patients and their parents was contingent on how well it was coordinated with clinical routines and family visits. Massage practitioners were attuned to these scheduling issues, and often tried to schedule their visits to the unit during the late afternoon lull in clinical activity.

*(R) What was the hardest thing about the massage study?*
*(P2) Scheduling…when they were ready, [my daughter] wasn't ready sometimes, or when [she] was ready, they were far away.*
* (P12) …it's evening, it's nighttime, so they're [massage practitioners] all gone. I would like to call them back [laughs].*

One parent felt that she had to “stand guard” at her child's hospital room door so that her massage would not be interrupted by hospital staff.

* (P13) …[the practitioner]* *coming in was very important to her…and that's when I had to step in and say, "She's getting a massage"…I had to be the keeper of the door. *

### 6.2. Massage during Periods of Acute Symptoms

During periods of acute discomfort or nausea, generally during the first week after chemotherapy, children varied in their response to massage, and some did not want it.

* (P6) I think the hardest thing was just being open to it when she really felt miserable.*
*(P17) …As he got more sick and was feeling worse, he wasn't able to have the massage therapist come in…So I would say at the beginning it's nice, and maybe at the end as they're getting back into normal life again, it's good. But there in the middle of the transplant, it's not so necessary.*
*(R) And what was the biggest barrier for giving a massage?*
*(P2) Basically, I do not know, if she's in deep pain or if she's not in the mood or she's sick and tired of the whole situation, you know?*

Several parents, however, reported that massage was particularly beneficial for their child during periods of acute discomfort.

*(P12) I remember one day she said she hardly to sleep. The whole body is miserable and tired. And I have a really gentle massage for her, but it help.*
*(P4) It's only whenever he feels nauseous and, yeah, vomiting, then he agree he want to have physical therapy [massage]. Most of all, he's always like not agree with the physical therapies.*

### 6.3. Length of Participation in the Study and Being “Won Over”

Most parents in the study were new to massage. Some were immediately convinced of the potential benefit of massage while others were initially doubtful about whether massage could be helpful for their child. However, through the course of the study most parents came to value massage as an important component of the healing process.

*(P21) Well, it's just something that he never really experienced before and was hesitant about in the beginning. But once they started, he looked forward to it every night that it was available.*
*(P13) … You tend to be in the hospital and you do not want extra people coming into your room. But once [the practitioner] came in and we realized the benefits that she was getting from it, J. welcome her in…*

## 7. Discussion

HCT has resulted in improved survival rates among children with certain cancers, immune deficiency syndromes, or bone marrow failure, but it can be an agonizing ordeal for patients and their families. Indeed, in 1998 bone marrow transplantation was described as “the most devastating treatment that the human body could be subjected to” [25]. Medical advances in intervening years have resulted in a less punishing regimen, but transplantation still remains arduous and disruptive of the lives of patients and their caregivers. This study contributes to a growing body of research suggesting that massage can help alleviate the distress associated with HCT among both patients and their family caregivers. The qualitative methods employed by this study reveal outcomes that were undetected—and undetectable—by the broader study's quantitative design, including several that have not yet been reported in the literature. New findings include reported benefits for patients in promoting sleep and providing symptom relief; benefits for parents in an increased sense of competence and respite from caregiving; and increased closeness between parent and child and a demonstrated willingness by parents to perform massage on their child

 According to parent caregivers, massage provided varying degrees of relief from pain, nausea, and other symptoms associated with HCT for most, but not all, participants. Nearly all parents reported that massage sessions facilitated a general state of comfort, relaxation, and pleasure for their child. These findings are consistent with a study that reported reduced pain and increased relaxation among pediatric cancer patients who received massage [6], and with the results of a study suggesting that massage for cancer patients promotes positive feelings, relaxation, and a sense of being special and cared for [26]. Whether these benefits are sustained over time is an important question for future research, given the long-term suffering among survivors of childhood cancer and family caregivers described in numerous studies [27–31].

 Massage helped children fall asleep. This finding is notable because HCT patients often find sleep elusive in the midst of chronic pain, nausea, and discomfort, yet sleep is rarely mentioned in the literature on massage and symptom management. Moreover, the promotion of comfort and sleep–states not easily reduced to dualistic conceptions of mind or body–suggest that the positive effects of massage are not physical *or* affective, but rather both simultaneously. This result is in line with previous research reporting that massage alleviates physical symptoms while also addressing the “existential suffering” associated with cancer and cancer treatments and improving patients' “quality of life” [20, 32, 33].

 It should not be assumed, however, that massage is relaxing and pleasurable for all patients at all times. As demonstrated by our results, patients' request for, and response to, massage varied widely throughout the different stages of HCT, with some patients declining massage during periods of acute pain and nausea, and others requesting massage at precisely these times. Massage enabled children undergoing HCT to become coagents in the therapeutic process, and most children embraced this agency without reserve—electing massage only when it suited them. While most children appeared to appreciate parent massage, one child felt unable to decline her father's massages when she did not want them. Individual patients' needs, and the specific ways in which family dynamics mediate these needs, should be carefully determined and addressed in any pediatric massage intervention so as to avoid coercion and the perception among patients that massage is obligatory.

 This study suggests that it is important to adapt a semistandardized massage protocol to the immediate, and always mutable, sensations and perceptions of individual patients; when it offers respite from long periods of tedium and inactivity; and when it does not interfere with treatment schedules and hospital routines. This is not to suggest that a carefully constructed protocol with clear instructions on acupressure and massage techniques for typical HCT-related symptoms is not important. However, a massage protocol that does not allow for flexibility within a semistandardized frame runs counter to the study's findings and may undermine some of massage's reported benefits.

 Parents in this study were, for the most part, amenable to learning massage techniques that they could perform for their child, and the resulting interactions tended to have a positive effect on parent-child relations and to mitigate parents' and patients' suffering. These findings are in alignment with a nascent body of research suggesting that learning to perform massage for a family member can alleviate caregivers' anxiety and feelings of helplessness [16, 17], and that caregiver-provided massage for chronically ill family members increases a sense of intimacy and connection between the provider and recipient of massage [19]. More broadly, these results offer support for an understanding of disease and healing as social and material processes that extend beyond the individual, physical body [34].

 Moreover, providing parents with a means of actively participating in their child's treatment and recuperation—and children with a sense of control over their bodies—may represent a shift in what one of the study's massage practitioners described as a hospital culture in which “the professionals are taking care of the children and the parents try not to get in the way.” Parents felt comforted and strengthened when they were able to alleviate their child's suffering through massage. What are the broader implications, then, of parents playing a more active role in supportive care? Future research on massage and its increasing inclusion in biomedical treatment regimes could help elucidate these processes.

 The success of parent-provided massage was dependent not only on parents' desire to help their child, but on the nature of the interaction between patient, parent, and massage practitioner, and on the development of trust and recognition within this triad. Massage is an inherently social practice, and for most of the children participating in the study, who was touching them was as important as which massage techniques were used. For example, in addition to the two teenage boys who decided not to enroll in the study, some older children, particularly boys, declined massage from their parent and/or from a massage practitioner (who were all women). These children's reluctance emerged postintervention in conversations between researchers and massage practitioners, rather than in interviews with participants, so the causes have not been explored in depth. In a study on massage for children with cancer, Post-White et al. reported no gender differences in response to massage, but 8 boys (over 30 percent of enrolled participants) failed to complete the study [6]. It is clear that more research is needed to examine how factors such as the child's age, sex, and ethnicity mediate the perception and experience of massage.

 The limitations of this investigation are primarily related to it being a small pilot study without data from direct interviews with patients. There are also limitations in the study's data collection methods. First, conducting interviews by telephone limited the interviewer's ability to establish rapport with participants. In addition, scheduling the interviews after parents and children returned home from the hospital meant that participants' perspectives were restricted to recall. Future research would be enriched by a series of in-depth, in-person interviews with both caregivers and patients over the course of treatment.

 While parent reports indicate that massage may offer some short-term symptom relief, and that teaching massage to a parent may increase her or his sense of self-efficacy in managing a child's symptoms, longer-term benefits for both symptom relief and parent-child relationships were beyond the scope of this study. Questions that could be explored in more detail in follow-up studies include whether massage mitigates long-term, posttreatment suffering associated with HCT among both patients and caregivers, including posttraumatic stress. If massage/acupressure can provide relief from symptoms and even provide pleasurable feelings, it may reduce parental feelings of helplessness in the face of their child's pain and discomfort, a key symptom of posttraumatic stress. [35] Future research could also investigate the extent to which massage influences children's perceptions of their bodies [36] as they struggle with chronic disease and prolonged, invasive treatments for these diseases.

## 8. Conclusion

This study suggests that parent- and practitioner-provided massage may reduce suffering associated with HCT among pediatric patients and their parent caregivers. According to parent caregivers, massage relieved symptoms associated with HCT, and promoted sleep, relaxation, and comfort for their child. The data suggest that massage also may enhance the experience of intimacy and connection between children and parents; offer relief from prolonged periods of social isolation, boredom, and anxiety that characterize life for families in the pediatric HCT unit; and enable both patients and parents to play a more active role in managing symptoms. As a simultaneously physical and social practice, massage as applied in the context of this study's hospital setting is a therapy whose effectiveness among children requires family support, practitioner flexibility, coordination with clinical routines, and affinity among those who perform and receive it.

## Figures and Tables

**Table 1:** Patient characteristics.

*N*	15

Demographics:	
Age (mean) [Range 5–18]	11.3

Sex	
Female	7
Male	8

Ethnicity	
White	8
Asian	3
Hispanic	3
Other	1

Diagnoses:	
Congenital or acquired bone marrow failure	5
Hematologic malignancy	5
Congenital immune deficiency	3
Solid tumor	2
Hemoglobinopathy	0

Transplant type:	
Autologous	3
Allogeneic	12
